# Effectiveness and Safety of Acupuncture for Post-Stroke Neurogenic Bladder: A Systematic Review and Meta-Analysis

**DOI:** 10.3390/medicina62040708

**Published:** 2026-04-07

**Authors:** Seungcheol Hong, Ji-cheon Jeong, Dong-jun Choi

**Affiliations:** 1 Phil Hospital of Korean Medicine Cheongju, Cheongju 28377, Republic of Korea; chol1992@naver.com; 2 Department of Internal Korean Medicine, College of Korean Medicine, Dongguk University, Goyang 10326, Republic of Korea; 3 Department of Internal Korean Medicine, Dongguk University Ilsan Oriental Hospital, Dongguk University Medical Centre, Goyang 10326, Republic of Korea

**Keywords:** neurogenic bladder, stroke, acupuncture, systematic review

## Abstract

*Objective*: This review is to systematically evaluate the clinical effectiveness and safety of acupuncture therapy for patients with post-stroke neurogenic bladder (PSNB). *Methods*: We included randomized controlled trials (RCTs) evaluating any type of acupuncture treatment for PSNB. Data extraction and quality assessment using Cochrane Risk of Bias 2.0 were performed. Meta-analysis was conducted for total effective rate (TER) and urodynamic parameters. *Results*: Ten RCTs involving 727 participants were included. Meta-analysis showed that acupuncture was associated with a reduction in residual urine volume (RUV), and increases in maximum cystometric capacity (MCC), and maximal urinary flow rate (Qmax). Acupuncture also showed a higher TER compared to control groups (RR = 1.23, 95% CI [1.15, 1.33], *p* < 0.001). However, wide 95% prediction intervals for urodynamic parameters indicated substantial uncertainty for future clinical applications. Adverse events were mild and infrequent, but only partly reported in two studies among included trials. *Conclusions*: Acupuncture as an adjunctive therapy suggests potential trends for improving clinical efficacy and urodynamic parameters in PSNB patients. However, no definitive conclusions can be drawn regarding its clinical efficacy or safety due to the very low certainty of evidence, high methodological heterogeneity, and limited reporting of adverse events. Therefore, these results must be interpreted with extreme caution. Further high-quality, large-scale randomized controlled trials with standardized protocols are essential to establish robust evidence regarding its clinical effectiveness and safety. *Protocol registration*: PROSPERO CRD42025643092.

## 1. Introduction

Post-stroke neurogenic bladder (PSNB), is a common complication resulting from ischemic or hemorrhagic lesions in the central nervous system (CNS), affecting 36% to 57% of stroke survivors. Neuro-urological dysfunction, defined as malfunctioning of the bladder and urinary sphincter, is characterized by symptoms related to bladder filling, emptying, or both [1]. The severity of this dysfunction depends on various factors, including the location, nature, extent, and progression of the neurological lesion [2,3]. PSNB manifests as urinary incontinence, retention, or frequency, all of which significantly impact patient recovery and quality of life (QoL) [4].

Although established diagnostic criteria specific to PSNB are still lacking, the condition is clinically well-recognized for its secondary nature following a stroke. Spasticity, a well-studied post-stroke complication, can severely impair bladder control, and PSNB may be exacerbated by associated deficits such as immobility or cognitive dysfunction [5,6]. Subtypes of PSNB manifest as either neurogenic urinary incontinence or neurogenic urinary retention. Urinary incontinence, the predominant symptom, usually results from bladder overactivity, urethral sphincter dysfunction, or a combination of both, and may persist for up to one year after stroke. It is correlated with large infarcts and has been observed in 73% of patients with hemorrhagic stroke and 64% with ischemic stroke. In contrast, urinary retention affects 13% and 52% of these patients, respectively [7,8]. Neurogenic urinary retention is typically characterized by detrusor underactivity or detrusor-sphincter dyssynergia, leading to impaired bladder emptying.

Conventional treatments include intermittent catheterization and bladder function training; however, these often face limitations regarding side effects or patient compliance. Rehabilitation strategies encompass assisted voiding techniques, such as the Credé maneuver, Valsalva maneuver, triggered reflex voiding, and pelvic floor muscle training (PFMT) [9,10]. Pharmacological options, including antimuscarinic drugs, beta-3 adrenergic receptor agonists, and cannabinoids, are used to alleviate symptoms [10]. Furthermore, Botulinum toxin type A injection into the bladder wall is recommended for adult patients with refractory neurogenic detrusor overactivity when behavioral, physiotherapeutic, and drug therapies prove ineffective or are poorly tolerated [3,11].

Neuromodulation techniques have also been explored. Transcutaneous electrical nerve stimulation (TENS) combined with exercise has shown significant improvements in spasticity and bladder function. While sacral nerve stimulation is an invasive treatment primarily approved for non-neurogenic lower urinary dysfunction, it may also benefit patients with neurogenic bladder [12,13]. Functional magnetic stimulation (FMS) remains a noninvasive but controversial option for improving urinary symptoms, though it is considered an add-on treatment for neurogenic bladder following spinal cord injury (SCI) [14].

Despite these conventional treatments, complementary interventions and management strategies for PSNB are essential to facilitate recovery and enhance QoL [3]. Acupuncture has emerged as a promising integrative approach to modulate the brain-bladder axis and improve autonomic control. Previous research has indicated that while evidence was insufficient to support the efficacy of acupuncture for neurogenic bladder specifically after SCI [15], electroacupuncture combined with moxibustion reduced the incidence of urinary tract infections, decreased residual urine volume, and increased bladder capacity in affected patients [16]. Although uncertainty remains regarding the superiority of acupuncture over sham procedures, it may slightly improve overactive bladder (OAB) symptoms compared to medication, with fewer minor adverse events [17]. Furthermore, acupuncture has shown comparable efficacy to conventional drug therapy for OAB, with combined therapy yielding even more favorable outcomes [18]. While evidence regarding its impact on MFR, BR, and UIR remains insufficient, acupuncture has effectively improved PVR and maximal cystometric capacity in other forms of neurogenic retention, such as that following hysterectomy [19].

Thus, while previous studies suggest the potential efficacy of acupuncture for improving PSNB, definitive evidence has yet to be established. This study aims to systematically evaluate the evidence on the effectiveness and safety of acupuncture for patients with PSNB based on randomized controlled trials (RCTs).

## 2. Materials and Methods

### 2.1. Protocol and Registration

The protocol for this review was registered in PROSPERO (ID: CRD42025643092) on 5 February 2025. This review adheres to the Preferred Reporting Items for Systematic Reviews and Meta-Analyses (PRISMA) 2020 guidelines [20] (Table S1).

### 2.2. Search Strategy

Two independent reviewers searched the following databases from their inception to March 2026: PubMed, EMBASE, the Cochrane Central Register of Controlled Trials (CENTRAL), AMED, CINAHL, CNKI, Wanfang, VIP, CiNii, and six Korean medical databases (KoreanTK, Oasis, RISS, DBpia, KISS, and ScienceON). The search terms included “Neurogenic bladder” and “Stroke,” with strategies tailored to the characteristics of each database (Appendix A).

### 2.3. Study Selection

Two independent reviewers performed study selection based on the predefined inclusion and exclusion criteria. Any disagreements were resolved through discussion or consultation with a third reviewer.

#### 2.3.1. Inclusion Criteria

Following the PICOS framework, we included RCTs that met the following criteria:

- Participants: Patients diagnosed with PSNB via clinical history, neurological deficits, and brain imaging, regardless of demographic characteristics, disease duration, or severity.
- Interventions: Acupuncture therapy, including manual acupuncture, warm needling, or electroacupuncture.
- Comparators: Conventional care (e.g., medication, bladder training, or catheterization), placebo/sham acupuncture, or no treatment.
- Outcomes: Studies reporting at least one primary or secondary outcome. (e.g., urodynamic parameters, total effective rate)
- Study Design and Time: Only RCTs without restrictions on publication date or language.

#### 2.3.2. Exclusion Criteria

Studies were excluded if they met any of the following:

- Non-PSNB Conditions: Neurogenic bladder caused by other etiologies, such as spinal cord injury, diabetes, or Parkinson’s disease.
- Ineligible Interventions: Trials that did not involve acupuncture or those comparing different types of acupuncture as the sole intervention (e.g., manual vs. electroacupuncture).
- Ineligible Study Designs: Case reports, non-randomized trials, crossover or retrospective designs, literature reviews, and animal studies.
- Incomplete Data: Studies where the full text was unavailable or data were insufficient for meta-analysis despite contacting the authors.

### 2.4. Data Extraction and Quality Assessment

Data on participants, interventions, and outcomes were independently extracted. The risk of bias was assessed using the Cochrane Risk of Bias 2.0 (RoB 2.0) tool [21]. Outcomes were categorized into “Low risk,” “Some concerns,” or “High risk” across five domains: bias arising from the randomization process, bias due to deviations from intended interventions, bias due to missing outcome data, bias in measurement of the outcome, and bias in selection of the reported result. Disagreements were resolved via consensus with a third reviewer.

### 2.5. Data Synthesis and Statistical Analysis

Meta-analysis was performed using Review Manager version 5.4. (The Cochrane Collaboration, 2020, London, UK). For binary outcomes, risk ratios (RR) or risk differences (RD) with 95% confidence intervals (CI) were calculated. For continuous outcomes, mean differences (MD) or standardized mean differences (SMD) with 95% CIs were used. A random-effects model was employed to account for anticipated clinical and methodological heterogeneity across trials. Statistical heterogeneity was assessed using the I^2^ test, with I^2^ > 50% indicating significant heterogeneity. For outcomes with high heterogeneity (I^2^ > 75%), we additionally calculated the 95% prediction interval (PI) using the formula provided by Borenstein et al. [22]

$$95\%PI=M\pm \left(t_{k-2}\times \sqrt{\tau^{2}+{SE}^{2}}\right)$$

This interval provides a range within which the effect of the intervention in a future study is expected to fall, offering a more robust interpretation of treatment effects in diverse clinical settings. Subgroup analysis and funnel plot analysis (for more than 10 studies) were planned to explore sources of heterogeneity and publication bias, respectively.

### 2.6. Certainty of Evidence

The certainty of evidence for each outcome was evaluated using the Grades of Recommendation, Assessment, Development, and Evaluation (GRADE) approach [23]. Evidence was categorized as high, moderate, low, or very low based on risk of bias, inconsistency, indirectness, imprecision, and publication bias.

## 3. Results

### 3.1. Results of the Search

**Figure 1:**
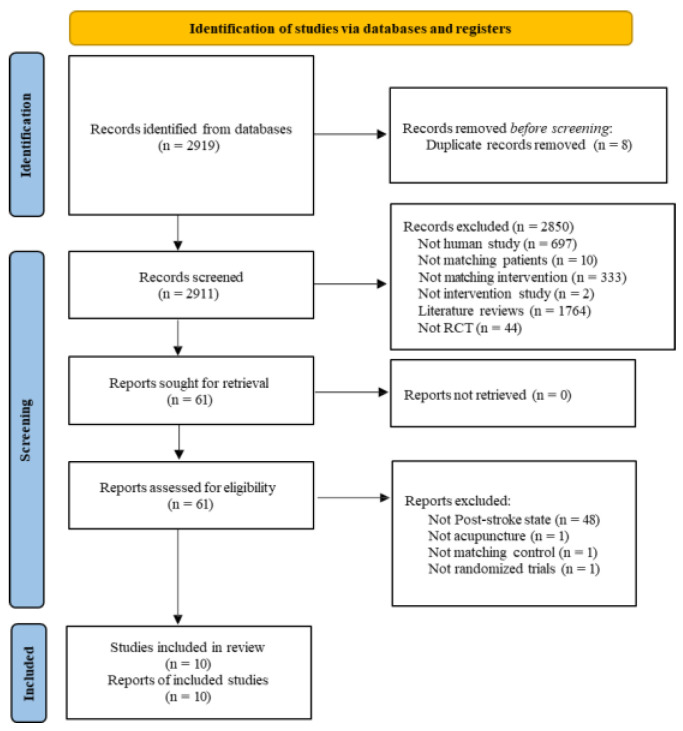
PRISMA Flow Diagram.

A total of 2919 articles were identified from the 15 electronic databases. After removing duplicate records and screening titles and abstracts, 61 articles were retrieved for full-text evaluation. Following a rigorous full-text review, 51 articles were excluded. (Figure 1 and Appendix B) Ultimately, 10 RCTs met the inclusion criteria and were included in the final qualitative and quantitative synthesis [24,25,26,27,28,29,30,31,32,33] (Table 1).

### 3.2. Characteristics of the Included Studies

A total of 727 patients with PSNB from ten RCTs were included, with no reported dropouts across the studies. While six trials included stroke patients without distinguishing between hemorrhage or infarction, none of the included RCTs restricted participants to a specific stroke subtype. Regarding PSNB subtypes, two trials [25,29] distinguished between urinary incontinence and retention, while three trials [26,30,31] focused exclusively on urinary retention; the remaining trials did not specify the subtype.

Sample sizes ranged from 60 to 96 participants per study, with an average of 72.7 patients. Only one trial [25] reported the methodology for sample size calculation. The duration of treatment varied from 20 days to 3 months.

Most trials evaluated acupuncture as an adjunct to bladder function training. Regarding the intervention types, seven trials applied manual acupuncture at conventional acupoints as an add-on treatment, two trials utilized electroacupuncture, and one trial employed plum-blossom needle tapping. Moxibustion was combined with acupuncture in three trials. None of the included trials utilized sham or placebo controls. Eight trials compared add-on acupuncture with conventional usual care (including intermittent catheterization, bladder function training, and pelvic floor exercises), while one trial used sacral nerve electrical stimulation, and another used herbal medication as the control group.

### 3.3. Outcome Assessment

Nine trials reported the total effective rate (TER) as a clinical outcome. Regarding urodynamic parameters, seven trials measured residual urine volume (RUV), while four trials assessed maximum cystometric capacity (MCC) and three trials reported the maximum urinary flow rate (Qmax) using B-mode ultrasound or urodynamic studies. Only two trials reported adverse events, while the remaining trials did not mention any safety-related outcomes.

### 3.4. Meta-Analysis: Urodynamic Parameters

#### 3.4.1. Residual Urine Volume (RUV)

**Figure 2:**
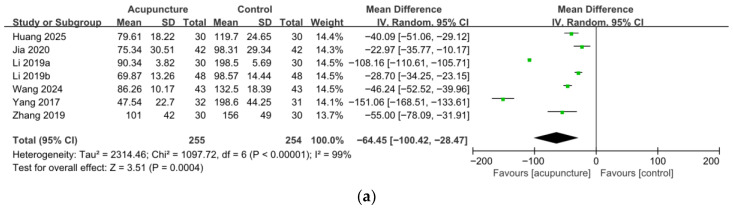

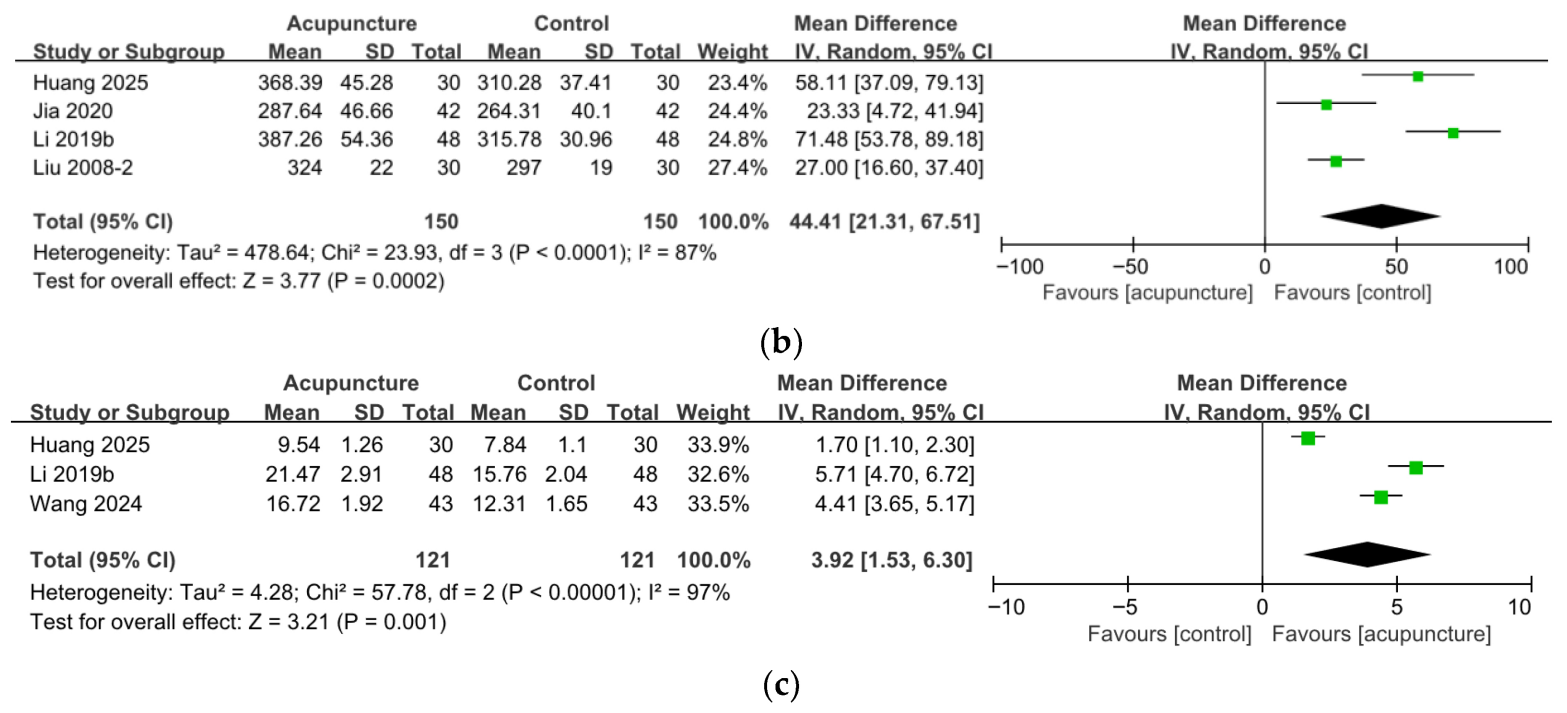
Forest plots of the effects of acupuncture on urodynamic outcomes: (**a**) Residual urine volume (RUV); (**b**) Maximal cystometric capacity (MCC); (**c**) Maximal urinary flow rate (Qmax) [25,26,27,28,29,30,31,33].

Seven trials involving 509 participants evaluated the effect of acupuncture on RUV. The pooled results showed a significant reduction in RUV in the acupuncture group compared to the control group (MD = −64.45 mL, 95% CI [−100.42, −28.47], *p* < 0.001). However, extreme statistical heterogeneity was observed (I^2^ = 99%). Due to this high heterogeneity, a 95% PI was calculated, ranging from −181.79 to 52.89 mL. This wide interval, including the null effect, suggests substantial uncertainty regarding the effect in individual future clinical settings. (Figure 2a).

#### 3.4.2. Maximal Cystometric Capacity (MCC)

Four trials involving 300 participants measured MCC. The meta-analysis revealed that acupuncture significantly improved MCC compared to the control group (MD = 44.41 mL, 95% CI [21.31, 67.51], *p* < 0.001). Significant heterogeneity was detected (I^2^ = 87%), and the 95% PI was determined to be −61.94 to 150.76 mL. While the pooled MD showed an increase of 44.41 mL, the broad prediction interval indicates that the effect may not be consistent across different clinical environments. (Figure 2b).

#### 3.4.3. Maximal Urinary Flow Rate (Qmax)

Three trials involving 242 participants reported Qmax. Acupuncture significantly increased Qmax compared to the control group (MD = 3.92 mL/s, 95% CI [1.53, 6.30], *p* = 0.001). Extreme statistical heterogeneity was observed (I^2^ = 97%), with a calculated 95% PI of −14.80 to 22.64 mL/s. This MD of 3.92 mL/s suggests a potential trend toward improvement, although the wide prediction intervals encompassing the null effect indicate significant uncertainty for future clinical applications in voiding velocity for PSNB patients. (Figure 2c).

### 3.5. Meta-Analysis: Total Effective Rate (TER)

**Figure 3:**
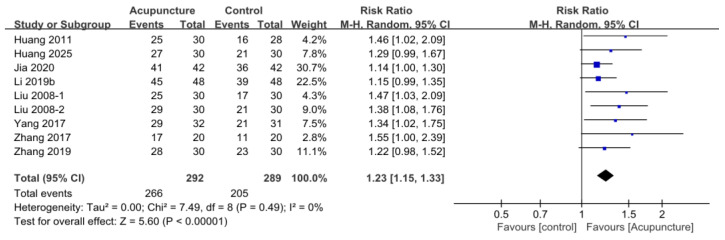
Forest plots of the effects of acupuncture on Total effective rate [24,25,26,28,29,31,32,33].

A meta-analysis of nine studies involving 581 participants demonstrated that the acupuncture group had a significantly higher TER compared to the control group (RR = 1.23, 95% CI [1.15, 1.33], *p* < 0.001). Statistical heterogeneity was negligible (I^2^ = 0%), reflecting a lack of statistical variation in the reported outcomes among the included trials. (Figure 3).

### 3.6. Safety

The safety profiles and occurrence of adverse events were assessed across the ten included studies. However, only two trials provided specific details regarding adverse reactions. Reported adverse events included mild skin burns associated with moxibustion and occasional needle-related bruising; no serious adverse events were reported. In the study by Huang 2025 [25], the intervention group reported one case (3.3%, 1/30) of a mild local skin burn following moxibustion, which recovered within 1–2 days after applying topical ointment. Conversely, the control group in the same study experienced three cases (10.0%, 3/30) of urinary tract infection (UTI) attributed to the catheterization procedure. Statistical analysis revealed no significant difference in adverse event incidence between the groups (*p* > 0.05). Zhang 2017 [32] explicitly stated that no serious adverse reactions were observed during the 4-week treatment period. The remaining eight trials did not provide specific data regarding adverse events.

### 3.7. Heterogeneity

Although this review included 727 patients from 10 trials, there was a high degree of heterogeneity regarding interventions (manual acupuncture, electroacupuncture, combined therapy), control treatments (usual care, rehabilitation, electrical stimulation, or herbal medicine), and outcome measurements (various urodynamic and voiding parameters). Except for the TER, all urodynamic outcomes exhibited significant statistical heterogeneity (I^2^ > 75%). This high heterogeneity suggests that the evidence for acupuncture’s efficacy in PSNB should be interpreted with caution, as conclusions may vary depending on the specific treatment modalities and outcome measures utilized.

### 3.8. Sensitivity Analysis

#### 3.8.1. Leave-One-Out Methods

To evaluate the robustness of our findings, a leave-one-out sensitivity analysis was performed (Appendix C). For the RUV, MCC, and TER, the exclusion of any single study did not significantly alter the overall pooled effect size or statistical significance, confirming the robustness of the results. Regarding Qmax, the pooled effect remained significant in most iterations; however, the exclusion of Wang 2024 [30] resulted in a loss of statistical significance (MD 3.68, 95% CI [−0.25, 7.61], *p* = 0.07), indicating that the current evidence for Qmax is highly sensitive to this specific trial.

#### 3.8.2. Excluding Electroacupuncture Treatment

To investigate the influence of intervention types, we conducted a sensitivity analysis by excluding studies that utilized electroacupuncture (EAT). The results for MCC (MD 50.98, 95% CI [21.78, 80.18], *p* < 0.001, I^2^ = 86%) and TER (RR 1.20, 95% CI [1.10, 1.30], *p* < 0.001, I^2^ = 0%) remained consistent with the primary analysis, suggesting that the results of primary analysis are not solely dependent on electrical stimulation.

### 3.9. Risk of Bias of Included Studies

**Figure 4:**
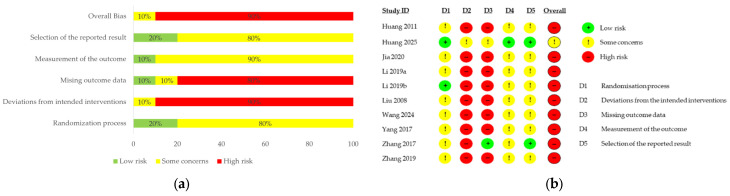
Risk of bias assessment using the Cochrane RoB 2.0 tool: (**a**) Risk of bias summary; (**b**) Risk of bias individual study assessments [24,25,26,27,28,29,30,31,32,33].

The methodological quality of the 10 RCTs was rigorously evaluated using the Cochrane Risk of Bias 2.0 (RoB 2.0) tool. Overall, the majority of studies were classified as having a “High Risk” of bias, primarily driven by the inherent difficulty of blinding in acupuncture trials. (Figure 4a,b).

Randomization Process (D1): While most studies mentioned “randomization,” only Huang 2025 [25] provided explicit details on central randomization and the use of opaque, sealed envelopes for allocation concealment. Li 2019b [28] also described a clear sequence generation using Excel. The remaining studies were rated as having “Some Concerns” due to insufficient detail regarding concealment protocols.

Deviations from Intended Interventions (D2): This domain represented the highest risk. Due to the nature of acupuncture and moxibustion, double-blinding was not feasible. While Huang 2025 mitigated this risk by blinding statistical analysts and outcome assessors, the other nine studies were rated as “High Risk” for potential performance bias.

Missing Outcome Data (D3): Although the included studies generally conducted analyses on the full randomized samples with seemingly negligible attrition rates, eight trials provided insufficient information regarding their dropout and withdrawal criteria. This lack of transparency resulted in a “High Risk” of bias for missing outcome data in those studies. Furthermore, while one trial [25] explicitly defined the criteria for dropouts in its methodology, it failed to report the actual dropout numbers in the results section, leading to “Some Concerns.” Only one trial [32] provided comprehensive reporting for both the predefined criteria and the final results of participant flow, warranting a “Low Risk” rating.

Measurement of the Outcome (D4): Huang 2025 [25] was rated as “Low Risk” for using blinded assessors. Other studies were rated as “Some Concerns”; however, the use of objective urodynamic parameters and ultrasonography—which are less susceptible to observer bias—somewhat mitigated this risk.

Selection of the Reported Result (D5): Eight of the included studies were rated as “Some Concerns”. Although their reported results appeared consistent with the predefined outcomes, the complete omission of dropout and withdrawal criteria raised suspicions regarding potential selective reporting bias. Given the lack of transparency in participant flow, the possibility of reported results being selectively chosen could not be entirely ruled out. Only two trials [25,32] were rated as “Low Risk,” as they provided sufficient methodological detail to dismiss concerns of reporting bias.

### 3.10. Publication Bias

Although 10 trials were included in the review, the maximum number of trials pooled for meta-analysis was nine (for TER). Consequently, funnel plot analysis and Egger’s test were not performed, as they are generally recommended only when at least 10 studies are available for a single outcome. However, it should be noted that all included trials were conducted and published in China. This geographical concentration suggests a potential for publication bias, as practitioners and patients in this region may have a more favorable disposition toward acupuncture, necessitating a cautious interpretation of the overall effects.

### 3.11. Certainty of Evidence

The certainty of evidence for each outcome was evaluated using the GRADE approach (Table 2).

1. Urodynamic Parameters (RUV, MCC, Qmax): The certainty of evidence for all three urodynamic outcomes was rated as Very Low. The evidence was downgraded based on the following criteria:
  - Risk of Bias: High risk of bias across all studies, particularly regarding blinding and allocation concealment.
  - Inconsistency: Presence of substantial statistical heterogeneity (I^2^ > 80%).
  - Imprecision: Although the 95% CI showed significant results, the 95% PI included the null value, suggesting potential for no effect in future clinical settings.
  - Other Considerations: High potential for publication bias due to all trials being published in China.
2. Total Effective Rate (TER): The certainty of evidence was rated as Very Low. Although heterogeneity was not serious, the evidence was downgraded due to risk of bias (lack of blinding and concealment), indirectness (diverse control interventions), and other considerations (potential bias from subjective and non-validated scale, and publication bias as all studies were conducted in China).

## 4. Discussion

Recent reviews suggest that acupuncture combined with conventional bladder training exerts an additive effect in restoring urinary function. From the perspective of Traditional Medicine, PSNB is often associated with “*Kidney Deficiency*” or “*Damp-Heat in the Bladder*”. Acupoints typically selected in the included trials—such as CV4, CV3, CV6, and SP6—aim to warm the Kidney and regulate the “*Water Passages*”. From a neurophysiological standpoint, stimulating these points is thought to modulate the pelvic and pudendal nerves, thereby facilitating the brain-bladder axis and enhancing autonomic control over micturition.

Our findings suggest that acupuncture may be potentially associated with the modulation of neural control in bladder function. However, since the included RCTs did not incorporate direct neurophysiological measurements and were characterized by a high risk of bias, these mechanistic pathways remains hypothetical and require further rigorous investigation with adequate control conditions. A notable observation in this review is the negligible statistical heterogeneity (I^2^ = 0%) observed in the TER. While this reflects a lack of statistical variation in the reported outcomes among the included trials, it should be interpreted with caution as it may also result from uniform research practices or shared systematic biases within a specific geographical region. Furthermore, the evidence remains insufficient to establish the superiority of acupuncture over conventional interventions due to the high heterogeneity of comparators and the absence of sham-controlled trials.

Despite the positive clinical outcomes, several limitations necessitate a cautious interpretation of the results.

First, significant statistical heterogeneity (I^2^ > 75%) was observed in urodynamic parameters, likely stemming from variations in study designs, specific acupuncture protocols (e.g., auricular vs. manual acupuncture), and the clinical diversity of participants, such as varying stroke types and disease severities. Although we planned subgroup analyses to explore these sources, they were not applicable due to the lack of granular data in the primary trials. Consequently, the pooled estimates for urodynamics should be interpreted as a potential trend rather than a definitive unified effect.

Second, the methodological quality of the included trials remains a significant concern. The complete absence of sham or placebo controls, and the resulting lack of blinding for both participants and practitioners, likely introduced a substantial positive bias. While there is ongoing debate regarding the physiological activity of sham acupuncture—suggesting it may not be a truly inert placebo—the complete absence of blinding in the included studies likely biased the results in favor of the intervention [34,35]. This potential for performance and detection bias, coupled with the “Very Low” certainty of evidence according to the GRADE assessment, underscores the necessity for more rigorous, multi-center trials with robust blinding protocols to confirm these findings.

Third, potential for missing outcome data and reporting bias was observed. Several trials failed to report any criteria for dropouts or withdrawals in their methodology, or omitted the results despite having predefined criteria (e.g., Huang 2025 [25]). Only one RCT [32] explicitly reported both the criteria and the corresponding results. Similarly, adverse events were only partially reported in two trials. Due to the limited and incomplete reporting, the definitive conclusion for safety of acupuncture for PSNB patients, which was a predefined secondary outcome in our protocol, cannot be drawn based on the data from included trials. This reflects a significant limitation in current reporting practices; while safety is an essential feature of clinical treatment, it is frequently neglected by acupuncture researchers and practitioners in certain clinical traditions. To establish the safety profile of acupuncture for PSNB, it is highly recommended that future trials must include safety outcomes as a standard requirement, even if no adverse events occur during the study period.

Lastly, a potential publication bias must be considered. As all included trials were conducted and published in China, where acupuncture is a deeply integrated part of the healthcare system, the findings and conclusions of this review must be extrapolated with caution to clinical settings with different geographical, cultural, and ethnic backgrounds. As acupuncture is deeply integrated into the Chinese healthcare system, participants in these regions may possess a more favorable disposition toward the intervention compared to those in Western or other cultural contexts. Consequently, the generalizability and external validity of these results to global populations remains to be validated through future international, multi-center trials.

Given the extreme statistical heterogeneity observed across urodynamic parameters, a leave-one-out sensitivity analysis was performed to evaluate the robustness of our findings. Our sensitivity analysis suggested that this heterogeneity is not attributed to a single outlier but likely arises from a complex combination of clinical factors, including variations in stroke severity, specific acupoint selections, and the duration of the intervention. Notably, for the Qmax outcome, the statistical significance was dependent on the inclusion of Wang 2024 [30]. This sensitivity underscores the need for larger-scale trials to confirm the definitive impact of acupuncture on urinary flow rates. While the consistent direction of effect across all sensitivity iterations might suggest a potential trend for the use of acupuncture as an adjunctive therapy, this should be viewed with significant caution. Furthermore, our analysis excluding electroacupuncture trials demonstrated that manual acupuncture alone yields significant improvements in both TER and MCC. This indicates that the manual acupuncture stimulation itself may be associated with modulating the neural control of the bladder in PSNB patients. However, as previously noted, given the low certainty of evidence and the absence of sham-controlled trials, such consistency may also reflect shared systematic biases across the included studies rather than a definitive reproducible clinical effect. Consequently, the impact of clinical heterogeneity remains a significant factor, and these outcomes should be interpreted as hypothesis-generating rather than confirmed clinical efficacy.

Therefore, the sources of heterogeneity are likely rooted in the clinical diversity of the participant pool—such as stroke duration, severity, baseline medications, and activities of daily living (ADL)—alongside the methodological variability of the acupuncture intervention and the inherent complexity of PSNB subtypes. Future RCTs must report data that control for these confounding factors to provide clearer evidence. Despite the presence of such inherent heterogeneity and the high I^2^ values observed in urodynamic parameters, the consistent direction and trend of the overall effect sizes in the sensitivity analysis suggest that the impact of this heterogeneity on the robustness of the meta-analysis remains limited.

Despite these limitations, this review provides a comprehensive overview of the current status of acupuncture research for PSNB. To improve the certainty of evidence, future research should implement standardized urodynamic testing, such as 24-h frequency-volume charts or automated flowmetry, to capture precise and objective changes in bladder behavior. Additionally, stratifying patient cohorts and reporting outcomes based on stroke subtypes—which could not be comprehensively synthesized in this review—would provide crucial data for a more rigorous analysis and discovery of the clinical effects of acupuncture in post-stroke sequelae. Given that the absence of sham/placebo controls in this review acts as a severe limitation in interpreting the specific clinical efficacy, rigorous double-blind, sham-controlled RCTs are required to isolate the effects of acupuncture from placebo responses. Furthermore, exploring the optimal “dose” of acupuncture—including frequency and duration—and ensuring transparent reporting of adverse events are essential steps toward filling the current evidence gaps. Ultimately, these methodological improvements will facilitate the development of standardized clinical guidelines, providing a more robust and safe therapeutic framework for the management of PSNB in global clinical practice.

## 5. Conclusions

Based on the current status of acupuncture research, acupuncture as an adjunctive intervention to conventional care suggests potential benefits for the management of PSNB. However, due to the very low certainty of evidence—characterized by the high risk of bias in the included trials, substantial statistical heterogeneity in urodynamic parameters, and a lack of standardized outcome measures like sham-controlled designs—no definitive conclusions can be drawn regarding its clinical efficacy. While acupuncture appeared to be associated with improvements in clinical effective rates and certain urodynamic outcomes in the analyzed studies, these results must be interpreted with extreme caution. Furthermore, no definitive conclusions can be drawn regarding the safety of acupuncture due to limited and incomplete data on adverse events. Although acupuncture might be considered a conditional add-on option in clinical settings, its routine application cannot be firmly recommended at this stage. While our synthesis of the available evidence suggests potential trends, the current quality of evidence is insufficient to support firm clinical recommendations. Therefore, rigorous, large-scale, and multi-center randomized controlled trials with standardized protocols and long-term follow-up are an absolute prerequisite before providing definitive clinical guidance on the therapeutic value of acupuncture for PSNB.

## Figures and Tables

**Table 1:** Study characteristics.

First Author /Year/ Country	Sample Size/ Age (year)	Patients	Intervention	Control Treatment	Duration	Outcomes	Summary of Results	Adverse Events
Huang 2011 China [24]	*n* = 58/ 67 (T), 65 (C)	PSNB patients	1. Electroacupuncture - BL23, BL28, BL31, BL32, BL33, BL34, with low frequency stimulation, maintained 30 min 2. Moxibustion - CV4, CV6, maintained 15 min 3. Routine treatment + BFT Once per day, 5 times/week, 3 weeks	1. Routine treatment 2. BFT	3 weeks	1. TER 2. UTI prevalence	1. TER: 83.4% (T), 57.1% (C) 2. UTI prevalence: 13.3% (T), 46.1% (C)	Not mentioned
Huang 2025 China [25]	*n* = 60/ 60 (T), 59 (C)	PSNB patients	1. Acupuncture - CV6, CV4, CV3, CV2, CV12, CV10, CV9, KI11, maintained 30 min 2. Moxibustion - The skin area between the umbilicus and the symphysis pubis, maintained 30 min 3. Routine treatment + BFT Once per day, 3 times/week, 4 weeks	1. Routine treatment 2. BFT	4 weeks	1. TER 2. Ultrasound, Urodynamics: Qmax, RUV, MCC, Pvesmax, PdetQmax 3. NBSS, LUTS, I-QoL	1. TER: 90% (T), 70% (C) 2. Ultrasound, Urodynamics - Qmax: 9.5 ± 1.26 (T), 7.8 ± 1.1 (C) - RUV: 79.6 ± 18.2 (T), 119.7 ± 24.7 (C) - MCC: 368.4 ± 45.3 (T), 310.3 ± 37.4 (C) - Pvesmax: 38.5 ± 5.4 (T), 31.9 ± 4.6 (C) - PdetQmax: 50.4 ± 8.0 (T), 40.3 ± 6.0 (C) 3. NBSS: 21.4 ± 3.9 (T), 31.3 ± 4.0 (C) - LUTS: 9.5 ± 1.4 (T), 15.3 ± 1.6 (C) - I-QoL: 98.3 ± 7.6 (T), 80.2 ± 6.1 (C)	1 case of mild local skin burn (T) 3 cases of UTI (C)
Jia 2020 China [26]	*n* = 84/ 62.0 (T), 62.3 (C)	NBUAS patients	1. Acupuncture - CV6, GV20, CV3, CV4, SP9, ST28, SP6, maintained 30 min, Once per day, 1 month 2. Medium-Frequency Pulse Electrical Stimulation: Abdomen area over the symphysis pubis, maintained 20 min, 1–2 times/day, 4–8 days 3. Control treatment	1. IC 2. BFT	1 month	1. TER 2. Ultrasound: RUV, MCC	1. TER: 97.6% (T), 85.7% (C) 2. Ultrasound - RUV: 75.3 ± 30.5 (T), 98.3 ± 29.3 (C) - MCC: 287.6 ± 46.7 (T), 264.3 ± 40.1 (C)	Not mentioned
Li 2019a China [27]	*n* = 60/ 55.9 (T), 55.8 (C)	PSNB patients	1. Acupuncture - CV6, GV20, CV3, CV4, SP9, ST28, SP6, N-CA-4, and Scalp acupuncture at MS6/MS7, MS12 - maintained 30 min, once per day, 6 time/week, 1 month 2. Intermittent Catheterization (IC)	1. BFT 2. Indwelling Catheter	1 month	1. Symptom remission time (day) 2. RUV 3. Barthel Index 4. Urinalysis (WBC)	1. Symptom remission time: 9.5 ± 0.3 (T), 15.7 ± 0.5 (C) 2. RUV: 90.3 ± 3.8 (T), 198.5 ± 5.7 (C) 3. Barthel Index: 78.3 ± 2.6 (T), 69.3 ± 3.0 (C) 4. WBC occurrence: 6.7% (T), 60.0% (C)	Not mentioned
Li 2019b China [28]	*n* = 96/ 46.1 (T), 45.3 (C)	PSNB patients	1. Acupuncture - BL20, BL39, GV3, SP6, BL37, BL57, BL32, BL13, BL23, maintained 30 min 2. Moxibustion - BL20, BL39, maintained 30 min 3. Control treatment Once per day, 4 times/week, 9 weeks	1. Routine treatment 2. SNS	9 weeks	1. TER 2. Voiding Parameters 3. Ultrasound, Urodynamics: RUV, MCC, Pdet, Qmax 4. NHISS, IPSS	1. TER: 93.8% (T), 81.3% (C) 2. Voiding Parameters - volume per voiding: 271.9 ± 92.2 (T), 259.5 ± 86.6 (C) - frequency of voiding: 6.3 ± 0.3 (T), 8.7 ± 0.7 (C) - frequency of urgency: 1.0 ± 0.3 (T), 2.1 ± 0.5 (C) 3. Ultrasound, Urodynamics - RUV: 69.9 ± 13.3 (T), 98.6 ± 14.4 (C) - MCC: 387.3 ± 54.4 (T), 318.5 ± 31.0 (C) - Pdet: 69.3 ± 9.1 (T), 57.5 ± 8.3 (C) - Qmax: 21.5 ± 2.9(T), 15.8 ± 2.0 (C) 4. NHISS: 3.9 ± 2.1 (T), 4.6 ± 1.4 (C) - IPSS: 4.4 ± 2.4 (T), 7.7 ± 3.6 (C)	Not mentioned
Liu 2008 China [29]	*n* = 120/ 66.6 (T), 65.6 (C)	PSNB patients	1. Electroacupuncture - Bilateral BL31, BL32, BL33, BL34, and BL35, low-frequency 40 Hz, maintained 30 min, Once per day, 6 times/week 2. Control treatment	Routine treatment	3 months	1. TER 2. Ultrasound: RUV, MCC 3. Grade of voiding parameters	1. TER - urinary retention: 83.3% (T), 56.6% (C) - urinary incontinence: 90% (T), 23.3%(C) 2. Ultrasound - MCC: 324 ± 22 (T), 297 ± 19 (C) 3. Grade - voiding frequency (Gr.1): 21/30 (T), 3/30 (C) - incontinence frequency (Gr.1): 18/30 (T), 2/30 (C)	Not mentioned
Wang 2024 China [30]	*n* = 86/ 67.7 (T), 67.4 (C)	NBUAS patients	1. Acupuncture - BL32, BL28, BL23, EX-HN1, GV20, CV6, ST36, CV4, SP6, SP9, LI11 - maintained 30 min, once per day, 6 times/week, 4 weeks 2. Control treatment	1. IC 2. BFT	4 weeks	1. Urodynamics 2. ERS 3. Grade of voiding parameters	1. Urodynamics - Qmax: 16.7 ± 1.9 (T), 12.3 ± 1.7( C) - RUV: 86.3 ± 10.2 (T), 132.5 ± 18.4 (C) - Pvesmax: 28.2 ± 3.3 (T), 23.7 ± 3.3 (C) 2. ERS - Caspase12: 89.5 ± 10.9 (T), 102.6 ± 13.2 (C) - GRP78: 10.4 ± 2.5 (T), 14.8 ± 3.6 (C) - CCAAT/EBPs: 879.7 ± 106.8 (T), 983.0 ± 125.1 (C) 3. Grade - Voiding function (Gr.0): 20/43 (T), 6/43 (C) - Urinary retention: 13/43 (T), 6/43 (C)	Not mentioned
Yang 2017 China [31]	*n* = 63/ 60 (T), 55 (C)	NBUAS patients	1. Acupuncture - LU11, LI1, ST45, SP1, HT9, SI1, BL67, KI1, PC9, TE, GB44, LR1 - withdrawn immediately without retention, Once per day, alternating between the sides each day, 20 days 2. Control treatment	1. Routine treatment 2. BFT 3. IC	20 days	1. TER 2. RUV	1. TER: 90.6% (T), 67.7% (C) 2. RUV: 47.5 ± 22.7 (T), 198.6 ± 44.3 (C)	Not mentioned
Zhang 2017 China [32]	*n* = 40/ 51.2 (T), 49.3 (C)	PSNB patients	Acupuncture - BL29, BL32, BL23, CV2, CV3, ST28 - For Facial Paralysis: ST4, ST6, ST44, LR3 - For Dysarthria: GV15, CV23, HT6, TE1 - maintained 15 min, once every other day, 4 weeks	Herbal medication - Astragali Radix, Angelicae Sinensis Radix, Paeoniae Radix Rubra, Chuanxiong Rhizoma, Persicae Semen, Carthami Flos - decoction, oral administration, 3 times per day	4 weeks	TER	TER: 85.0% (T), 55.0% (C)	No events
Zhang 2019 China [33]	*n* = 60/ 63.8 (T), 65.6 (C)	PSNB patients	1. Acupuncture - BL23, BL24, BL26, BL27, BL28, BL35, BL31–BL34, tapping by Plum-blossom needle 2. Moxibustion - CV8, CV4, CV6, CV3, 3–5 min Once per day, 2 months	1. Routine treatment 2. BFT 3. IC	2 months	1. TER 2. Ultrasound 3. Voiding parameters 4. Grade of symptoms	1. TER: 93.3% (T), 76.7% (C) 2. Ultrasound - RUV: 101 ± 43 (T), 156 ± 49 (C) 3. Voiding parameters - volume of voiding: 178 ± 48 (T), 154 ± 53 (C) - frequency of voiding: 6.0 ± 2.2 (T), 9.1 ± 2.4 (C) - frequency of incontinence: 2.3 ± 1.9 (T), 4.2 ± 2.5 (C) 4. Grade - urinary symptoms: 8.9 ± 3.1 (T), 12.8 ± 4.2 (C)	Not mentioned

BFT, bladder function training; C, control group; ERS, endoplasmic reticulum stress; Gr, Grade; IC, intermittent catheterization; MCC, maximum cystometric capacity; NBUAS, neurogenic bladder urinary retention after stroke; PSNB, post-stroke neurogenic bladder; Qmax, maximal urinary flow rate; RUV, residual urine volume; SNS, Sacral Nerve Electrical Stimulation; T, treatment group; TER, total effective rate; UTI, urinary tract infection; WBC, white blood cell.

**Table 2:** Summary of findings.

Certainty Assessment							Effect		Mean Difference (95% CI)	Grade Quality of Evidence	Importance
Outcomes	Participants (Studies)	Risk of Bias	Inconsistency	Indirectness	Imprecision	Other Considerations	Relative (95% CI)	Absolute (95% CI)			
RUV	*n* = 509 (7 RCTs)	Serious ^a^	Serious ^b^	Not serious	Serious ^d^	Serious ^e^			MD −64.45 mL (−100.42 to −28.47)	⨁◯◯◯ Very low	Important
MCC	*n* = 300 (4 RCTs)	Serious ^a^	Serious ^b^	Not serious	Serious ^d^	Serious ^e^			MD 44.41 mL (21.31 to 67.51)	⨁◯◯◯ Very low	Important
Qmax	*n* = 242 (3 RCTs)	Serious ^a^	Serious ^b^	Not serious	Serious ^d^	Serious ^e^			MD 3.92 mL/s (1.53 to 6.30)	⨁◯◯◯ Very low	Important
TER (overall)	*n* = 581 (9 RCTs)	Serious ^a^	Not serious	Serious ^c^	Not serious	Serious ^e^^,f^	RR 1.23 (1.15 to 1.33)	162 more per 1000 (106 to 233 more)		⨁◯◯◯ Very low	Important

CI, confidence interval; MCC, maximum cystometric capacity; MD, mean difference; Qmax, maximal urinary flow rate; RR, relative risk; RUV, residual urine volume; SMD, standardized mean difference; TER, total effective rate. Explanations. ^a^. All studies had high risk of bias, such as lack of blinding, concealment. ^b^. Presence of substantial heterogeneity. (I^2^ > 80%). ^c^. Compared with various control interventions. ^d^. Although 95% CI is significant, the 95% PI includes the null value, indicating potential for no effect in future clinical settings. ^e^. All studies were published in China. ^f^. The outcome was based on subjective and non-validated scale.

## Data Availability

The data that support the findings of this study are available from the corresponding author upon reasonable request.

## Supplementary Materials

The following supporting information can be downloaded at: https://www.mdpi.com/article/10.3390/medicina62040708/s1, Table S1: PRISMA statement checklist.

## Abbreviations

The following abbreviations are used in this manuscript:

ADL	Activities of Daily Living
BFT	Bladder Function Training
CENTRAL	Cochrane Central Register of Controlled Trials
CI	Confidence Interval
CNS	Central Nervous System
EAT	Electroacupuncture/Electroacupuncture Treatment
FMS	Functional Magnetic Stimulation
GRADE	Grades of Recommendation, Assessment, Development, and Evaluation
IC	Intermittent Catheterization
I-QoL	Incontinence Quality of Life
IPSS	International Prostate Symptom Score
LUTS	Lower Urinary Tract Symptoms
MA	Manual Acupuncture
MCC	Maximum Cystometric Capacity
MD	Mean Difference
NBSS	Neurogenic Bladder Symptom Score
OAB	Overactive Bladder
PFMT	Pelvic Floor Muscle Training
PI	Prediction Interval
PRISMA	Preferred Reporting Items for Systematic Reviews and Meta-Analyses
PSNB	Post-Stroke Neurogenic Bladder
Qmax	Maximal Urinary Flow Rate
Qol	Quality of Life
RCT	Randomized Controlled Trial
RoB 2.0	Cochrane Risk of Bias tool, version 2.0
RR	Risk Ratio
RUV	Residual Urine Volume
SCI	Spinal Cord Injury
SMD	Standardized Mean Difference
SNS	Sacral Nerve Electrical Stimulation
TENS	Transcutaneous Electrical Nerve Stimulation
TER	Total Effective Rate
UTI	Urinary Tract Infection

## Appendix A. Search Strategy

### Appendix A.1. Medline (via Pubmed) Search Strategy

**Group**	**Search Terms and Strategy**	**Targets**
Population	#1. Urinary Bladder, Neurogenic [MeSH] #2. Stroke [MeSH] OR infarction [MeSH] OR Intracranial hemorrhage [MeSH] #3. Stroke$ [TIAB] OR CVA [TIAB] OR cerebrovascular [TIAB] OR cerebral [TIAB] OR intracranial [TIAB] OR brain [TIAB] OR infarction [TIAB] OR ischemia [TIAB] OR hemorrhage [TIAB] OR haemorrhage [TIAB] #4. #1 AND (#2 OR 3)	PSNB
Intervention	#5. Medicine, Chinese Traditional [MeSH] OR acupuncture therapy [MeSH] OR acupuncture [MeSH] #6. Acupuncture [TIAB] OR electroacupuncture [TIAB] OR pharmacoacupuncture [TIAB] OR acupressure [TIAB] OR acupoint* [TIAB] #7. Herb$ [TIAB] OR herbal$ [TIAB] OR decoction$ [TIAB] OR botanic$ [TIAB] #8. TCM [TIAB] OR traditional Chinese [TIAB] OR oriental [TIAB] OR traditional Korean [TIAB] OR traditional Japanese [TIAB] OR kampo [TIAB] OR CAM [TIAB] OR complementary [TIAB] OR alternative [TIAB] #9. #5 OR #6 OR #7 OR #8	Acupuncture & Traditional Medicine
(P & I)	#10. #4 AND #9	
Study design	#11. Randomized controlled trials as topic [MeSH] OR Controlled Clinical Trials as Topic [MeSH] OR Clinical Trials as Topic [MeSH] #12. clinical trial* [TIAB] OR controlled trial* [TIAB] OR randomized [TIAB] OR RCT* [TIAB] OR case report* [TIAB] OR systematic [TIAB] #13. in vitro [TIAB] OR rat [TIAB] OR mice [TIAB] OR mouse [TIAB] OR rabbit [TIAB] #14. (#11 OR #12) NOT #13	Clinical Trials
Total combined	#15. #10 AND #14 (Total results: 704)	Final Selection

### Appendix A.2. Embase (via Ovid) Search Strategy

**Group**	**Search Terms and Strategy**	**Targets**
Population	#1. urinary bladder, neurogenic/exp #2. Stroke/exp #3. infarction/exp #4. Intracranial hemorrhage/exp #5. #1 or #2 or #3 or #4	PSNB
Intervention	#6. Drugs, Chinese herbal/exp #7. Herbal Medicine/exp #8. Plants, Medicinal/exp #9. Medicine, Chinese Traditional/exp #10. acupuncture/exp #11. (acupuncture$ OR electroacupuncture$ OR pharmacoacupuncture$ OR pharmacopuncture$ OR acupressure$ OR acupoint$).mp #12. ((traditional Korean medicine) OR (traditional Chinese medicine) OR (traditional oriental medicine) OR (Kampo medicine) OR (alternative medicine) OR (complementary medicine) OR herb$ OR herbal$ OR decoction$ OR botanic$).mp #13. #6 or #7 or #8 or #9 or #10 or #11 or #12	Acupuncture & Traditional Medicine
Study design	#14. Randomized Controlled Trials as Topic/exp #15. controlled clinical trials as topic/exp #16. (randomized controlled trial$ OR controlled clinical trial$ OR randomized$ OR randomly$ OR placebo OR clinical trial$or controlled trial$).mp #17. #14 or #15 or #16	Clinical Trials
Total combined	#18. #5 and #13 and #17 (Total results: 1867)	Final Selection

## Appendix B. List of Excluded Studies with Reasons

1. Guo LQ. Clinical Study on Electro Acupuncture Associated with Neurogenic Bladder Dysfunction after Spinal Cord Injury Treated Intermittent Catheterization [Master’s thesis]: Henan University of Chinese Medicine; 2015. Reason: Not matching patients (SCI)
2. Kim KW. Comparison of lower urinary tract symptoms and urodynamic findings according to the presence of prostatitis in patients with benign prostatic hyperplasia [Master’s thesis]: Chonnam National University; 2002. Reason: Not matching patients (Not NB)
3. Kim YW. Intravesical anhydrous C-fiber blocker and cold water-induced urodynamic study in hyperreflexic neurogenic bladder [Master’s thesis]: Yonsei University; 2004. Reason: Not matching patients (SCI)
4. Kim JH. Comparison of the effects of intravesical instillation of atropine and oxybutynin for neurogenic bladder in spinal cord injury patients [Master’s thesis]: Chonnam National University; 2000. Reason: Not matching patients (SCI)
5. Kim HS. Cut-off values for bladder outlet obstruction in women using pressure-flow study [Master’s thesis]: University of Ulsan; 2000. Reason: Not matching patients (Not NB)
6. Ryu KH. Results of urine culture and antibiotic susceptibility tests according to voiding methods in spinal cord injury patients [Master’s thesis]: Konkuk University; 2011. Reason: Not matching patients (SCI)
7. Li N. Warm Acupuncture Combined with Bladder Function Training in the Treatment of Neurogenic Bladder after Spinal Cord Injury Research [Master’s thesis]: Hubei University of Chinese Medicine; 2014. Reason: Not matching patients (SCI)
8. Li YQ. Clinical Research on Electro-acupuncture Combined with Magnetic Stimulation of The Sacral Nerve Roots for Treatment of The Neurogenic Bladder after Spinal Cord Injury [Master’s thesis]: Hubei University of Chinese Medicine; 2015. Reason: Not matching patients (SCI)
9. Li HL, Guo YH, Yin HN, editors. Survey of Electric Acupuncture Treatment on Neurogenic Bladder after Spinal Cord Injuries in Recent Five Years. 2017 World Acupuncture and Moxibustion Academic Conference; 2017; Beijing, China. Reason: Not RCTs (Survey)
10. Meng ZX, Wang T, Yin ZL, Wang JB. Clinical research of electroacupuncture combined with transperineal injection of BTX-A for neurogenic bladder after spinal cord injury. Chinese Acupuncture & Moxibustion. 2015;35(01):17–20. Reason: Not matching patients (SCI)
11. Bae HS. Effect of rectal electrical stimulation on neurogenic bowel in spinal cord injury patients [Master’s thesis]: Yonsei University; 2002. Reason: Not matching outcomes (Neurogenic bowel)
12. Shin MJ. Bladder compliance and overactivity in low-capacity neurogenic bladder [Master’s thesis]: Pusan National University; 2009. Reason: Not matching patients (SCI)
13. Wang N, Feng QV, Lin XW, Zhang XL, Zhao J. Effect of acupuncture combined with Jingui Shenqi Pills on patients with neurogenic bladder and urine retention after spinal cord injury. World Journal of Integrated Traditional and Western Medicine. 2020;15(03):524–7+31. Reason: Not matching patients (SCI)
14. Wang SF. Clinical study on warm needle acupuncture at conventional acupoints combined with the theory of Sanjiao for post-stroke neurogenic bladder [Master’s thesis]: Hebei University of Chinese Medicine; 2023. Reason: Not matching control (Acupuncture control)
15. Wang XY, Zhao RR, Sun XH. Clinical Study on Herbal Hot Compress Packs Combined with Acupoint Application for Diabetic Neurogenic Bladder. Journal of Practical Traditional Chinese Medicine. 2025;41(11):2113–5. Reason: Not matching patients (Diabetic NB)
16. Wang HL, Ding YY, Yang GM, Wang SL, Zhang LJ. Clinical Study on Intervention of Chinese Medicine Treatment and Nursing for Neurogenic Bladder After Spinal Cord Injury. Journal of New Chinese Medicine. 2019;51(03):252–4. Reason: Not matching patients (SCI)
17. Wang W. A clinical study on treating diabetic neurogenic bladder by acupuncture the Baliao point. Clinical Journal of Chinese Medicine. 2011;3(02):14+6. Reason: Not matching patients (Diabetic NB)
18. Wang W. Clinical Study of Oral Chinese Medicine combined with Moxibustion Treatment of Diabetic Neurogenic Bladder. China Journal of Chinese Medicine. 2014;29(12):1729–30. Reason: Not matching patients (Diabetic NB)
19. Wang XG, Wang DL, Wang R. Clinical Study of Fire Needling ‘Urine Three-Acupoint’ in Treatment of NB after SCI. Journal of Clinical Acupuncture and Moxibustion. 2023;39(06):39–43. Reason: Not matching patients (SCI)
20. Niu L, Li YJ, Peng JF, Qin HW, Guo N, Sun YB, et al. Study on the Efficacy of Wenyang Tongli Prescription Acupoint Application on Neurogenic Urinary Retention after Spinal Cord Injury. Journal of Emergency in Traditional Chinese Medicine. 2022;31(06):993–6+1008. Reason: Not matching patients (SCI)
21. Yin P, Zheng HM, Tang KM, Chen YL. The Clinical Research Progress of Acupuncture Treatment on Neurogenic Bladder. Guiding Journal of Traditional Chinese Medicine and Pharmacy. 2016;22(17):62–5. Reason: Not RCTs (Review article)
22. Lee SK. Prediction of Quinolone Antibiotics and ESBL-producing Resistant E. coli in Community-acquired Urinary Tract Infection Patients [Master’s thesis]: Chungbuk National University; 2014. Reason: Not matching patients (Not NB)
23. Lee SB. Understanding of Neurogenic Bladder Using Intravenous Urography in Spinal Cord Injury Patients [Master’s thesis]: CHA University; 2007. Reason: Not matching patients (SCI)
24. Lee JW, Lee EJ, Shin BC, Lee MS, Lim SM, Cho CS, et al. Clinical Practice Guideline for Acupuncture Treatment of Post-stroke Urinary Retention. Journal of Korean Medicine. 2016;37(1):1–9. Reason: Not RCTs
25. Zhu KX, Jin S, Shao WF, Zhu JF, Ni HL. Clinical Study on Routine Rehabilitation Training and Electroacupuncture Combined with Warming-Needle Moxibustion for Neurogenic Bladder Urinary Retention After Spinal Cord Injury. New Chinese Medicine. 2022;54(01):164–7. Reason: Not matching patients (SCI)
26. Zhou JM, Fan DS. Clinical Study of Warm Acupuncture Combined with Lipoid Acid Injection for Treatment of Diabetic Neurogenic Bladder. Chinese Journal of Information on Traditional Chinese Medicine. 2016;23(02):45–8. Reason: Not matching patients (Diabetic NB)
27. Zhu Y, Li N, Li JA, Wu Y, Tang XM. Clinical Research of Neurogenic Bladder Retention of Urine after Spinal Cord Injury Dealt with Acupuncture and Rehabilitation. Chinese Journal of General Practice. 2010;8(12):1495–7. Reason: Not matching patients (SCI)
28. Zeng QM, Zheng ZH. A clinical study on treating diabetic neurogenic bladder by acupoint sticking. Clinical Journal of Chinese Medicine. 2016;8(16):34–5+7. Reason: Not matching patients (Diabetic NB)
29. Zhan YH, Liu XG, Sun SJ, Tu QM. Clinical Study on Modified Buyang Huanwu Tang Combined with Tongdu Tiaoshen Acupuncture for Neurogenic Bladder After Spinal Cord Injury. New Chinese Medicine. 2022;54(15):36–40. Reason: Not matching patients (SCI)
30. Shen J, Wang WY, Kan ZH, Qi YJ, Shen Z, Bi CH. Clinical Study on Acupuncture-moxibustion plus Intermittent Catheterization in Treating Neurogenic Bladder After Spinal Cord Injury. Shanghai Journal of Acupuncture and Moxibustion. 2018;37(10):1187–90. Reason: Not matching patients (SCI)
31. Liu G. Clinical Study on Acupuncture Treatment of Neurogenic Bladder [Master’s thesis]: Anhui University of Chinese Medicine; 2017. Reason: Not RCTs
32. Liu Q. Clinical Study on the Treatment of Uninhibited Neurogenic Bladder after Apoplexy with Na Zi Method and Electroacupuncture [Master’s thesis]: Heilongjiang University of Chinese Medicine; 2020. Reason: Not matching control (Acupuncture control)
33. Hua XJ, Liu G, Wang T, Li SR. Clinical Study on Treatment of Urination Disorder after Stroke by Moxibustion Combined with Acupoint Application of Chinese Medicine. Shandong Journal of Traditional Chinese Medicine. 2017;36(05):390–2. Reason: Not matching intervention (Not acupuncture)
34. Lyu TT, Lyu JW, Jiang C, Li H, Liu HR. Clinical Study of Diabetic Neurogenic Bladder Treated by Electro-Acupuncture Nerve Stimulation. Journal of Clinical Acupuncture and Moxibustion. 2019;35(01):34–7. Reason: Not matching patients (Diabetic NB)
35. Sun WJ, Liu CM, Wang L, Bai JM, Duan HJ, Ren YF. Effect of heat sensitive moxibustion on neurogenic bladder after spinal cord injury clinical randomized controlled study. Lishizhen Medicine and Materia Medica Research. 2022;33(11):2688–90. Reason: Not matching patients (SCI)
36. Zhang HT, Wei NY, Liang XN, Wei QL. Clinical observation on the treatment of neurogenic bladder after spinal cord injury by thunder-fire moxibustion combined with electroacupuncture. Journal of Modern Medicine & Health. 2020;36(23):3769–72. Reason: Not matching patients (SCI)
37. Yang RC, Mao ZF, Yang JH. Clinical Study of Wenshen Liniao Prescription Combined with Electropuncture for Neurogenic Bladder Urinary Retention After Spinal Cord Injury. New Chinese Medicine. 2023;55(19):109–14. Reason: Not matching patients (SCI)
38. Luo J, Yang H, Xie QW. Clinical Study on Acupuncture Combined with Rehabilitative Training for Neurogenic Bladder After Spinal Cord Injury. Journal of New Chinese Medicine. 2020;52(23):111–4. Reason: Not matching patients (SCI)
39. Tan YQ. The Clinical Study in Treating Neurogenic Bladder Dysfunction with Acupuncture on Scalp and Body [Master’s thesis]: Heilongjiang University of Chinese Medicine; 2009. Reason: Not matching patients (SCI)
40. Deng HW, Wei W, Chen YF, Li YB. Clinical Research of Electroacupuncture at Baliao Acupoint in the Treatment of Neurogenic Bladder after Spinal Cord Injury. Journal of Emergency in Traditional Chinese Medicine. 2017;26(09):1631–3. Reason: Not matching patients (SCI)
41. Zheng YF. Clinical Studies on Moxibustion and Acupuncture for the Treatment of Multiple Sclerosis of Neurogenic Bladder. Liaoning Journal of Traditional Chinese Medicine. 2015;42(07):1329–30. Reason: Not matching patients (Multiple Sclerosis)
42. Zheng J, Liu H, Guo HY, Zhang PF, Pan SJ, Gao ZZ. Survey of Acupuncture and Moxibustion Treatment on Neurogenic Bladder after Spinal Cord Injuries in Recent Ten Years. Journal of Liaoning University of Traditional Chinese Medicine. 2013;15(07):203–5. Reason: Not RCTs (Review article)
43. Chen YS, Kong XY, Jia JR. Clinical Research of Acupuncture for Neurogenic Bowel Dysfunction Due to Spinal Cord Injury. Journal of New Chinese Medicine. 2017;49(04):148–50. Reason: Not matching outcomes (Neurogenic bowel)
44. Chen SY, Zhang AG, Wang HF, Pan SL, Chen SQ. Clinical Study on Electroacupuncture at Baliao Points Combined with Long Snake Moxibustion at Du Mai for Neurogenic Bladder After Spinal Cord Injury. New Chinese Medicine. 2024;56(01):150–4. Reason: Not matching patients (SCI)
45. Wei HL, Ren YF, Zhang ZL, Huang XM, Li B. Efficacy differences between electroacupuncture and moxibustion for neurogenic bladder after spinal cord injury: a randomized controlled trial. Chinese Acupuncture & Moxibustion. 2023;43(09):1036–41. Reason: Not matching control
46. Ma SH. Clinical Study on the Treatment of Neurogenic Bladder by Transcutaneous Electrical Nerve Stimulation Combined with acupuncture and moxibustion. Smart Healthcare. 2025;11(26):63–5+9. Reason: Not matching patients (SCI/Others)
47. Dean GE, Long C. Updates in the management of the overactive bladder in patients with myelomeningocele. Current Urology Reports. 2011;12(6):413–8. Reason: Not matching patients (Myelomeningocele)
48. Kennelly M, Cruz F, Herschorn S, Abrams P, Onem K, Solomonov VK, et al. Efficacy and Safety of AbobotulinumtoxinA in Patients with Neurogenic Detrusor Overactivity Incontinence Performing Regular Clean Intermittent Catheterization: Pooled Results from Two Phase 3 Randomized Studies (CONTENT1 and CONTENT2). European Urology. 2022;82(2):223–32. Reason: Not matching intervention (Botulinum toxin)
49. Lee BB, Toh SL, Ryan S, Simpson JM, Clezy K, Bossa L, et al. Probiotics [LGG-BB12 or RC14-GR1] versus placebo as prophylaxis for urinary tract infection in persons with spinal cord injury [ProSCIUTTU]: a study protocol for a randomised controlled trial. BMC Urol. 2016;16:18. Reason: Not matching patients (SCI)
50. Min JH. Effect of transcutaneous spinal cord magnetic stimulation on detrusor overactivity in patients with spinal cord injury [Master’s thesis]: Pusan National University; 2022. Reason: Not matching patients (SCI)
51. Sasaki CT, Leder SB. Therapeutic effects of acupuncture for neurogenic dysphagia—A randomized controlled trial: Comment. Dysphagia. 2013;28(1):118. Reason: Not matching patients (Dysphagia, not NB)

## Appendix C. Sensitivity Analysis

**Table A1:** Leave-one-out analysis.

Outcome First Author/Year	Sample Size	RR/MD	95% CI	*p*-Value	I^2^ Value	Interpretation
Residual urine volume (RUV)						
Original	509	−64.45	−100.42, −28.47	*p* < 0.001	99%	baseline
Excluding:						
Huang 2025 [25]	449	−68.54	−108.14, −28.93	*p* < 0.001	100%	Robust
Jia 2020 [26]	425	−71.36	−109.90, −32.82	*p* < 0.001	99%	Robust
Li 2019a [27]	449	−56.47	−56.47, −32.32	*p* < 0.001	97%	Mildly decreased effect
Li 2019b [28]	413	−70.50	−107.22, −33.78	*p* < 0.001	99%	Robust
Wang 2024 [30]	423	−67.55	−109.92, −25.19	*p* = 0.002	99%	Robust
Yang 2017 [31]	446	−50.26	−88.92, −11.61	*p* = 0.01	100%	Decreased significance
Zhang 2019 [33]	449	−65.96	−105.02, −26.89	*p* < 0.001	100%	Robust
Maximum cystometric volume (MCC)						
Original	300	44.41	21.31, 67.51	*p* < 0.001	87%	baseline
Excluding:						
Huang 2025 [25]	240	40.27	12.17, 68.37	*p* = 0.005	90%	Robust
Jia 2020 [26]	216	51.43	21.50, 81.37	*p* < 0.001	90%	Increased effect
Li 2019b [28]	204	34.80	16.32, 53.27	*p* < 0.001	74%	Decreased effect and heterogeneity
Liu 2008 [29]	240	50.98	21.78, 80.18	*p* < 0.001	86%	Increased effect
Maximal urinary flow rate (Qmax)						
Original	242	3.92	1.53, 6.30	*p* = 0.001	97%	baseline
Excluding:						
Huang 2025 [25]	182	5.02	3.75, 6.29	*p* <0.001	76%	Improved significance and effect
Li 2019b [28]	146	3.04	0.39, 5.70	*p* = 0.02	97%	Decreased effect
Wang 2024 [30]	156	3.68	−0.25, 7.61	*p* = 0.07	98%	Non-significant
Total effective rate (TER)						
Original	581	1.23	1.15, 1.33	*p* < 0.001	0%	baseline
Excluding:						
Huang 2011 [24]	523	1.22	1.14, 1.32	*p* < 0.001	0%	Robust
Huang 2025 [25]	521	1.24	1.14, 1.34	*p* < 0.001	0%	Robust
Jia 2020 [26]	497	1.28	1.17, 1.39	*p* < 0.001	0%	Robust
Li 2019b [28]	485	1.26	1.16, 1.37	*p* < 0.001	0%	Robust
Liu 2008 [29]	461	1,21	1.12, 1.31	*p* < 0.001	0%	Robust
Yang 2017 [31]	518	1,23	1.14, 1.32	*p* < 0.001	2%	Robust
Zhang 2017 [32]	541	1.22	1.14, 1.32	*p* < 0.001	0%	Robust
Zhang 2019 [33]	521	1.24	1.14, 1.35	*p* < 0.001	8%	Robust

**Table A2:** Sensitivity analysis excluding electroacupuncture.

Outcome	Sample Size	RR/MD	95% CI	*p* -Value	I^2^ Value	Interpretation
**Maximum cystometric volume (MCC)**						
*Original*	300	44.41	21.31, 67.51	*p* < 0.001	87%	*baseline*
*Excluding EAT*	240	50.98	21.78, 80.18	*p* < 0.001	86%	Increased effect
**Total effective rate (TER)**						
*Original*	581	1.23	1.15, 1.33	*p* < 0.001	0%	*baseline*
*Excluding EAT*	403	1.20	1.10, 1.30	*p* < 0.001	0%	Robust
